# Supplementary material for: MRI-Derived Body Composition and Breast Cancer Risk in Postmenopausal Women: UK Biobank Study
Source: Cancers (Basel). 2025 Dec 18;17(24):4036. doi: 10.3390/cancers17244036 (PMC12731068; doi:10.3390/cancers17244036)
Supplement: Supplementary file 1 [file cancers-17-04036-s001.zip › cancers-4015048-supplementary.pdf]

Supplementary Material

**Supplementary Table S1.** Characteristics of study participants by MRI scan status.

| Variables | One or no MRI scan | At least two MRI scans | p-value |
|---|---|---|---|
| Age at recruitment | 77.3 (5.5) | 75.5 (5.2) | 0.678 |
| Age at menarche | 12.6 (2.9) | 12.6 (2.6) | 0.863 |
| Age at menopause | 46.3 (13.6) | 47.3 (12.2) | 0.591 |
| Index of multiple derivation | 14.0 (3.0) | 15.3 (2.7) | 0.096 |
| Ethnicity | | | |
| White | 133653 (89.3) | 14307 (91.3) | <0.001 |
| Others | 16014 (10.7) | 1364 (8.7) | |
| Smoking status | | | |
| Never | 86284 (57.7) | 9650 (61.6) | <0.001 |
| Former | 50617 (33.8) | 5297 (33.8) | |
| Current | 12766 (8.5) | 724 (4.6) | |
| Alcohol drinker status | | | |
| Never | 9700 (6.5) | 522 (3.3) | <0.001 |
| Former | 5886 (3.9) | 345 (2.2) | |
| Current | 134081 (89.6) | 14804 (94.5) | |
| Hormone replacement therapy | | | |
| No | 79186 (52.9) | 8628 (55.1) | <0.001 |
| Yes | 70480 (47.1) | 7043 (44.9) | |
| Bilateral oophorectomy | | | |
| No | 141110 (94.3) | 15024 (95.9) | <0.001 |
| Yes | 8556 (5.7) | 647 (4.1) | |

**Supplementary Table S2**. Cut-offs of tertiles of various body composition parameters.

| Tertiles of body composition phenotypes | Cutoffs |
|---|---|
| Visceral adipose tissue | |
| Tertile 1 | 1.85 |
| Tertile 2 | 3.12 |
| Tertile 3 | 12.27 |
| Subcutaneous adipose tissue | |
| Tertile 1 | |
| Tertile 2 | 6.06 |
| Tertile 3 | 8.56 |
| | 23.16 |
| Total adipose tissue | |
| Tertile 1 | 8.10 |
| Tertile 2 | 11.74 |
| Tertile 3 | 32.63 |
| Fat free muscle volume | |
| Tertile 1 | 7.41 |
| Tertile 2 | 8.59 |
| Tertile 3 | 13.29 |
| Muscle fat infiltration | |
| Tertile 1 | 7.15 |
| Tertile 2 | 8.60 |
| Tertile 3 | 28.43 |

**Supplementary Table S3.** Assessment of fit of models using Schoenfeld residuals.

| body composition parameters | Chi-square | df | p-value |
|---|---|---|---|
| Subcutaneous adipose tissue | 5.20 | 2 | 0.39 |
| Visceral adipose tissue | 1.09 | 2 | 0.58 |
| Total adipose tissue volume | 2.8 | 2 | 0.27 |
| Fat-free muscle volume | 4.74 | 2 | 0.19 |
| Muscle fat infiltration | 3.07 | 2 | 0.38 |
| Body composition phenotypes | 4.70 | 3 | 0.58 |

**Supplementary Table S4.** Descriptive Characteristics of Body Composition Variables.

| Body composition measures | Mean | SD | Min | 25th | Median | 75th | Max |
|---|---|---|---|---|---|---|---|
| Fat-free muscle volume | 7.8928 | 1.1372 | 0.0000 | 7.1658 | 7.8496 | 8.5855 | 13.2852 |
| Total adipose tissue volume | 10.3534 | 4.3951 | 0.2467 | 7.1822 | 9.8533 | 12.9220 | 32.6310 |
| Subcutaneous adipose tissue volume | 7.6505 | 3.1667 | 0.2199 | 5.4175 | 7.2626 | 9.4370 | 23.1632 |
| Visceral adipose tissue volume | 2.7029 | 1.4898 | 0.0268 | 1.5681 | 2.4445 | 3.5503 | 12.2729 |
| Muscle fat infiltration | 8.1089 | 1.8745 | 3.4964 | 6.8064 | 7.8153 | 9.0917 | 28.4266 |
